# Targeting Wnt Signaling via Notch in Intestinal Carcinogenesis

**DOI:** 10.3390/cancers11040555

**Published:** 2019-04-18

**Authors:** Elke Kaemmerer, Min Kyung Jeon, Alexander Berndt, Christian Liedtke, Nikolaus Gassler

**Affiliations:** 1 Institute of Pathology, RWTH Aachen University, 52074 Aachen, Germany; ekaemmerer@ukaachen.de; 2 Division of Medical Oncology, Yonsei Cancer Center, Yonsei University College of Medicine, Seoul 03722, Korea; mkjeon@yuhs.ac; 3 Section Pathology, Institute of Legal Medicine, University Hospital Jena, 07747 Jena, Germany; alexander.berndt@med.uni-jena.de; 4 Department of Medicine III, RWTH Aachen University, 52074 Aachen, Germany; cliedtke@ukaachen.de

**Keywords:** cancer, caspase 8, intestine, lncRNA, Notch, targeting, Wnt

## Abstract

Proliferation and differentiation of intestinal epithelial cells is assisted by highly specialized and well-regulated signaling cascades. The Wnt pathway, which is one of the fundamental pathways in the intestine, contributes to the organization of proliferative intestinal crypts by positioning and cycling of intestinal stem cells and their derivatives. The Wnt pathway promotes differentiation of intestinal secretory cell types along the crypt-plateau and crypt-villus axis. In contrast to the Wnt pathway, the intestinal Notch cascade participates in cellular differentiation and directs progenitor cells towards an absorptive fate with diminished numbers of Paneth and goblet cells. Opposing activities of Notch and Wnt signaling in the regulation of intestinal stem cells and the enterocytic cell fate have been elucidated recently. In fact, targeting Notch was able to overcome tumorigenesis of intestinal adenomas, prevented carcinogenesis, and counteracted Paneth cell death in the absence of caspase 8. At present, pharmacological Notch inhibition is considered as an interesting tool targeting the intrinsic Wnt pathway activities in intestinal non-neoplastic disease and carcinogenesis.

## 1. Introduction

Throughout the intestinal tract, a high throughput of surface lining epithelial cells is found. Type and differentiation of these cells vary along the small and large intestine and contribute to the establishment of the crypt-villus axis (CVA), as well as crypt-plateau axis (CPA). The epithelial renewal is tightly regulated by important signaling pathways and proteins, including Wnt, Notch, bone morphogenetic protein (BMP), and epidermal growth factor (EGF). Among these, the Wnt and Notch signaling cascades constitute primary driving activities assisting intestinal stem cells (ISC) in maintenance and proliferation, as well as post-mitotic cells in differentiation. The Wnt pathway is essential for establishment and maintenance of the intestinal crypt, a prototype stem cell compartment [1].

It has been suggested that spatial secretion of Wnt proteins is essential in growth control and patterning during development of multicellular structures. The function of Wnt proteins depends on acting either as a short or long distance paracrine activator of gene transcription [2]. However, the molecular mechanisms behind induction and maintenance of Wnt gradients in physiology, as well as in intestinal carcinogenesis, remain incompletely understood [3].

Besides its crucial role in tissue physiology, the Wnt pathway is found to be activated in several human diseases, including cancer. Mutations in the *APC* gene constitute principal mechanisms by which the Wnt signaling is aberrantly activated, driving colorectal carcinogenesis [4]. At present, the canonical β-catenin-dependent pathway is the best characterized Wnt signaling cascade and its aberrant activity is found in both the non-hypermutated microsatellite stable (MSS) and the hypermutated microsatellite instability (MSI) colorectal cancers (CRCs). In contrast to the canonical Wnt signaling, the roles of other pathways are not well characterized in colorectal carcinogenesis. This includes the planar cell polarity (PCP), c-Jun N-terminal protein kinases (JNK), the receptor-like tyrosine kinase (RYK), receptor tyrosine kinase-like orphan receptor (ROR), and the protein kinase C/calcium (PCK/Ca^2+^) pathways.

Due to its important role in carcinogenesis, there has been growing interest since the late 1990s in developing therapeutic agents targeting the Wnt signaling pathway. At present, four classes of inhibitors are defined according to their specific targets: (i) generic inhibitors, (ii) inhibitors targeting the Wnt-receptor complex, (iii) inhibitors targeting the β-catenin destruction complex, and (iv) inhibitors targeting the nuclear/transcription factor complex. In these categories, several substances and therapeutic agents are available for modulating the Wnt signaling activity. Yet, a clinically approved drug is still missing [4]. There are few FDA-approved generic drugs that have been shown to non-specifically modulate Wnt pathway activities. In this category, non-steroidal anti-inflammatory drugs (NSAIDs) are important therapeutics probably repressing the canonical Wnt-dependent transcription via inhibition of cyclooxygenase 2 (COX2) [5,6].

Some putative inhibitors of the Wnt signaling act more specifically at the protein porcupine, which is located in the endoplasmic reticulum (ER) as a member of the membrane-bound O-acyltransferase family (MBOAT). The enzyme is involved in N-glycosylation [7,8,9] and lipidation of Wnt proteins, resulting in their hydrophobicity and affinity to membranes [10]. The functional association and contribution of fatty acid metabolizing enzymes to the synthesis and modification of Wnt proteins are of high importance and build the molecular link between cell intrinsic fatty acid metabolism and the organization and coordination of cell and tissue homeostasis [11]. At present, porcupine is assumed to be unique for Wnt lipidation, because compensatory mechanisms have not been identified yet [12,13]. The “inhibitors of Wnt production” (IWP), discovered in 2009, are a class of small molecules that probably specifically suppress Wnt protein production at the level of porcupine.

While directly targeting the Wnt signaling pathway in CRC is not yet possible, alternative strategies are tested. Since a significant crosstalk between Notch and Wnt signaling exists, targeting Notch is probably an interesting approach to modulate Wnt pathway activities. In this review, the current understanding in the Wnt and Notch signaling pathways, their crosstalk, and pharmacological targeting of Wnt via Notch using Dibenzazepine is summarized in the context of intestinal carcinogenesis.

## 2. Wnt Signaling Pathway

**Figure 1:**
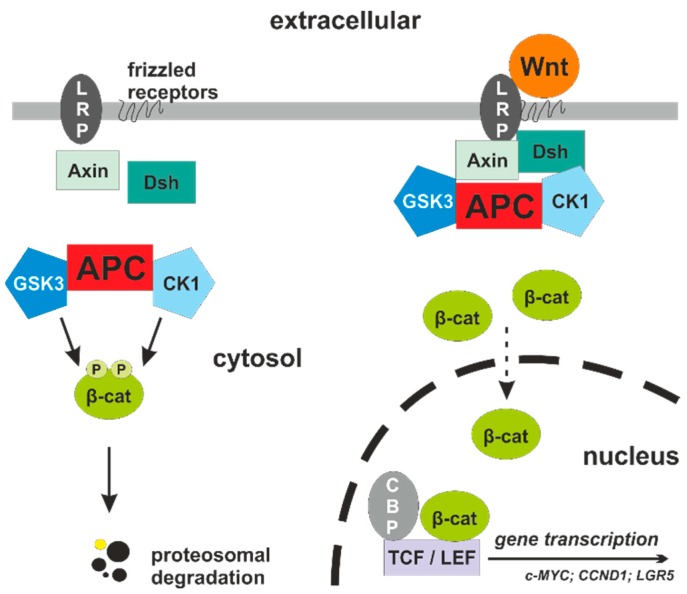
Canonical Wnt signaling pathway. Left: In the absence of Wnt ligands, the multi-protein complex with APC, GSK3, and CK1 cause phosphorylation and subsequent proteosomal degradation of β-catenin. Right: Upon binding of a Wnt protein ligand, the phosphorylation activity of the protein complex is inhibited and β-catenin accumulates in the cytoplasm and translocates to the nucleus with activation of gene transcription. APC: adenomatous polyposis coli; β-cat: β-catenin; CBP: cAMP-response element-binding protein; CK1: casein kinase 1; Dsh: Disheveled; GSK3: glycogen synthase kinase 3; LRP: low-density lipoprotein-related protein.

The canonical Wnt pathway is a receptor-based hierarchical signaling cascade for impulse transduction regulated by phosphorylation and ubiquitin-mediated degradation of proteins (Figure 1). The central messenger protein is cytoplasmic β-catenin. In the absence of any Wnt activation, the core protein complex of Axin, APC, GSK3, and CK1 phosphorylates β-catenin [14]. In a next step, the E3 ubiquitin ligase β-TrCP is recruited to the complex for ubiquitination and subsequent proteasomal degradation. The negative regulation of Wnt signaling by Axin und APC includes direct mechanisms described above and an indirect effect. In this activity, APC enhances the export of nuclear β-catenin to the cytoplasm, reducing the amount of β-catenin for the active transcriptional complex with T cell factor (TCF)/lymphoid enhancer-binding factor (LEF). The direct binding of β-catenin to APC is important in blocking the β-catenin interaction with TCF/LEF [15,16].

Upon secretory Wnt protein engagement to the membrane—anchored receptors Frizzled and low-density lipoprotein-related protein 5/6 (LRP5/6), the β-catenin destruction complex is inhibited by its recruitment to the membrane and subsequent dissociation of β-TrCP from the complex [17]. The cytosolic scaffold protein Disheveled (Dsh) acts as a connector of interaction between the Wnt receptors and the Axin complex. The destruction activity of the complex is inhibited by interfering with Axin oligomerization and enhancement of Wnt receptor signalosomes [18,19]. The loss of degradation and ubiquitination of β-catenin results in accumulation of cytosolic β-catenin with subsequent translocation into the nucleus. Assisted by other co-activators, nuclear β-catenin is able to displace the repressor Groucho from the TCF/LEF complex, resulting in the start of transcription. At present, there are several Wnt/TCF target genes identified, including *c-MYC*, *CDKN1A*, *LGR5*, and *CCND1*.

Aberrant activation of the Wnt signaling pathway is a frequent initiator and important driver of CRC development [20]. Physiologically at the bottom of crypts, Wnt signaling is activated supporting stem cell niche and tissue homeostasis by forming a driving gradient [1]. Mutations in the *APC* gene with loss of its tumor suppressor activities are crucial to initiate adenoma formation, as described by the bottom-up model. In this view, the important molecular event in tumor progression from adenoma to CRC is an irreversible inactivation of the *APC* gene and stepwise acquisition of other gene mutations, such as in *KRAS*, *TP53*, *SMAD4*, and *PI3KCA* [21]. Epigenetic silencing is an additional mechanism involved in activation of the Wnt pathway. Silencing of Wnt antagonists, such as DKK1, DKK3, and SFRP1, as well as of core proteins in the destruction complex, such as APC or Axin, has been described [22,23]. In CRCs characterized by MSI, the mutation rate of the entire genome, as well as of Wnt pathway components, is increased due to the defective DNA mismatch repair system.

Loss of function mutations in the *APC* gene are associated with the development of intestinal adenomas in mice as well as in humans. Importantly, the degree of polyposis depends on the mutated region in the gene. High polyposis penetrance is associated with germ-line mutations of the *APC* gene at codons 1061 and 1309 in patients suffering from familial adenomatous polyposis (FAP). In sporadic CRCs, the hotspot mutational region is frequently found between codons 1309 and 1450, associated with premature protein truncation caused by frameshift and nonsense mutations [21]. The non-functional *APC* gene is associated with permanent intrinsic activation of Wnt signaling due to loss of β-catenin ubiquitination. The biallelic loss of APC by secondary loss of heterozygosity mutations is somatic rather than germ line [4]. It has been hypothesized that in addition to the APC activities in the Wnt signaling cascade, the APC molecule may be crucial for tumor initiation by functions in microtubule binding, mitosis, and regulation of the cytoskeleton [24].

In CRCs and other neoplastic diseases with a de-regulated Wnt pathway, inhibitory proteins other than APC are affected by mutation or modification. In such cases, disturbed intrinsic activity of Wnt signaling is APC independent. Important mutated molecules are the negative regulators of the Wnt signaling cascade, such as Axin1, Axin2, WTX (synonym AMER1), as well as RNF43 and its homolog ZNRF3 [17,25,26,27]. In addition, several studies have described activating mutations affecting *CTNNB1*, *RSPO*, and other genes [28,29,30,31]. The group of RSPO proteins bind to the extracellular domains of LGR4/5 and RNF43, resulting in increased surface frizzled (FZD) receptors, with subsequent RNF43 clearing [32,33].

## 3. Notch Signaling Pathway

**Figure 2:**
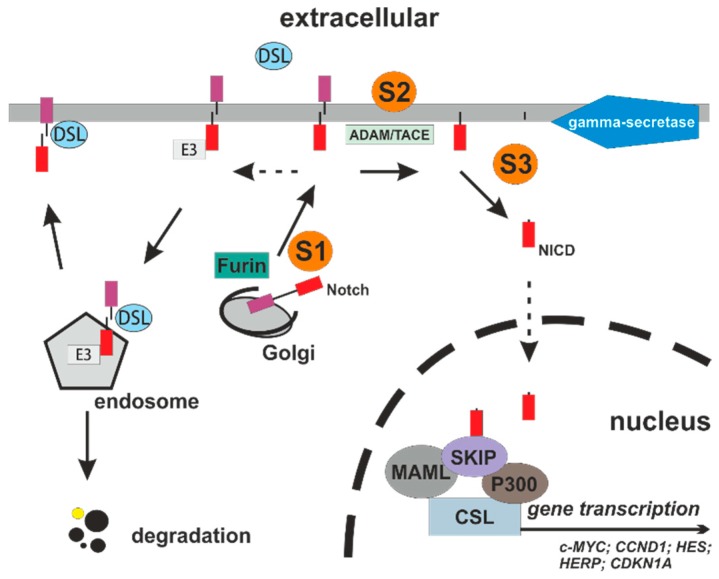
Canonical Notch signaling pathway. After (S1) cleavage by furin or furin-like convertases the heterodimeric Notch receptor translocates to the cell membrane. Right: Following ligand binding, proteolytic cleavage mediated by ADAM/TACE results in the release of NEC (S2) and additional cleavage at the transmembrane region by the γ-secretase complex creates the NICD (S3), which is able to translocate into the nucleus with subsequent activation of gene transcription. Left: Alternatively, after ubiquitination by E3-ligases, internalization of Notch to endosomes is possible. From endosomes, Notch can be recycled to the plasma membrane, pooled by cis-inhibition by DSL or degraded in the lysosome. ADAM/TACE: metalloproteinase containing protein; CSL: C-promoter binding factor-1 (CBF1 in humans, also known as CSL); DSL: Delta/Serrate Ligand; E3: E3-ligases; MAML: mastermind-like; NEC: Notch extracellular; NICD: Notch intracellular domain; SKIP: Ski-interacting protein.

Similar to the Wnt signaling, the Notch pathway includes canonical and non-canonical signaling activities important for cell–cell communication and short-range juxtacrine communications [34]. Notch is involved in several tissue forming activities, such as cell self-renewal, migration, and maintenance of stem cell features. There are two defined pathways: CSL-dependent signaling, which is the most important variant (CBP/RBP-jκ in vertebrates, suppressor of hairless in Drosophila, and Lag-1 in *Caenorhabditis elegans*) and Deltex protein signaling transduction [35]. Deltex generally acts as a positive regulator of Notch function by prevention of Notch receptors from degradation. The conserved pathway consists of four Notch receptors and five DSL ligands (JAG1 and 2, as well as delta-like 1, 3, and 4) as single transmembrane proteins, which act without involvement of second messenger molecules [36]. Following ligand binding, the Notch intracellular domain (NICD) is released from Notch proteins by two proteosomal cleavages mediated by ADAM family proteases (S2 cleavage generates NEC) and by γ-secretase (S3 cleavage generates NICD). It is able to translocate to the nucleus forming a transcriptional activator complex with the DNA-bound protein CSL and the transcription co-activator MAML [37,38]. This complex displaces repressor proteins, such as SMRT and SHARP, to upregulate transcription of the *Hairy enhancer of split* (*HES*) gene and *Hes-related repressor protein* (*HERP*) gene families of transcription repressors. Other genes upregulated by Notch signaling are *ERBB2*, *CCND1*, *NOTCH4*, and *NFIB2* [39,40]. Importantly, the NICD transmits Notch transcriptional activities and represses genes encoding the Notch ligands. The Notch is a target for phosphorylation, as well as protein ubiquitination and degradation (Figure 2).

The strength of Notch signaling is important for cell fate decisions mediated by an inverse correlation of Notch activity and expression of Notch ligands. This allows cells to become Notch ligand competent following the principle of lateral inhibition, where activation of Notch represses production of Notch ligands. This mechanism amplifies and stabilizes the stochastic initial differences in Notch signaling between two equivalent adjacent cells, rapidly driving them towards opposite fates [41]. Aberrations in the stability of NICD by mutations in its C-terminal domain are crucial in the tumorigenesis of different solid and hematological neoplasia [34].

In the intestine, the genetic program upon Notch activation varies in the cell types with stem cell behavior. In the case of stem/progenitor cells at the crypt, basal proliferation is triggered from Notch activation, whereas transit amplifying cells react with differentiation to an absorptive phenotype.

The activities of non-canonical Notch are ill-defined and their contribution to physiology, pathophysiology, or carcinogenesis is not clear. However, non-canonical signaling might be the rule rather than the exception [38]. The principle of non-canonical Notch is apparently Notch activation independent of a ligand or any protease. In some cases the expression of Notch target gene expression is apparently independent of CSL. Similar to the canonical Notch pathway, the non-canonical Notch signaling activates target genes belonging to the *HES*/*HEY* family of transcription factors [40]. However, there is some evidence for a transcription-independent activity of Notch that relies on the nuclear localization of the NICD. The protein Ataxia Telangiectasia Mutated, which is recruited to sites of DNA damage with “DNA damage response”, is negatively regulated by nuclear Notch with an inverse correlation [42].

## 4. Notch-Wnt Crosstalk

**Figure 3:**
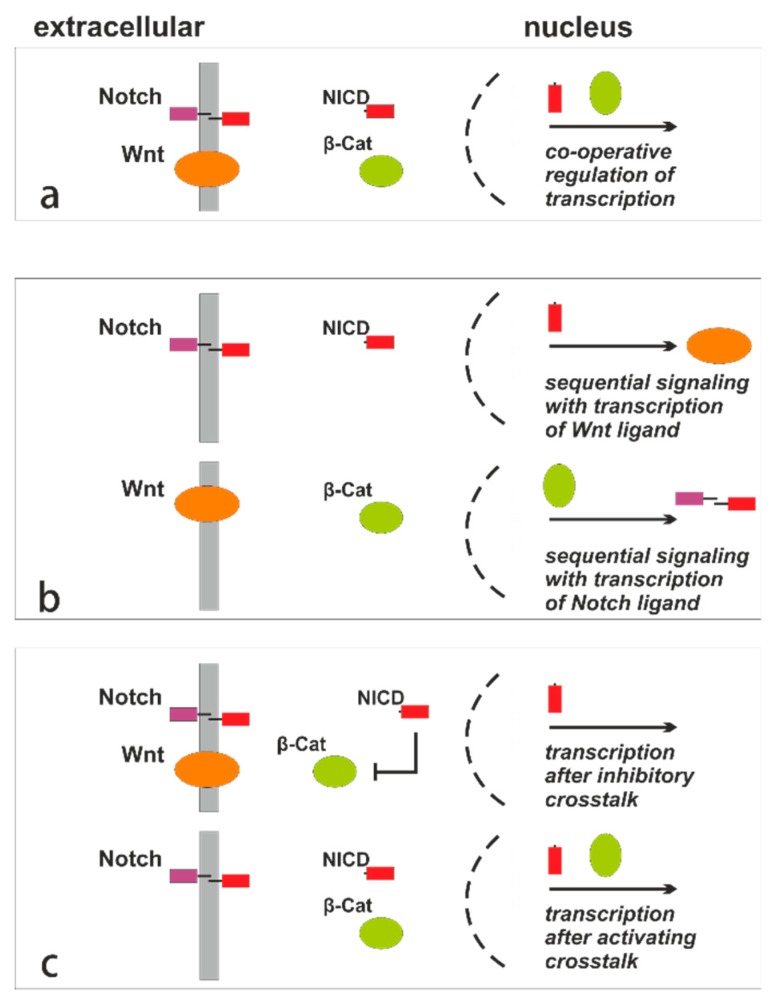
Variants of the Notch-Wnt crosstalk. (**a**) Co-operative and synergistic regulation of gene transcription when both pathways are active. (**b**) Sequential signaling is initiated when one active pathway promotes transcription of the ligand of the other pathway (top: active Notch signaling promotes expression of Wnt ligands; below: active Wnt signaling promotes expression of Notch ligands). (**c**) Examples of direct molecular crosstalk (top: both pathways are activated, but β-catenin is inhibited by NICD (inhibitory crosstalk); below: the Notch pathway is activated and co-activates intracellularly the Wnt cascade (activating crosstalk).

In general, Notch and Wnt pathways have distinct and opposing effects on cell-fate decisions and outcomes with driving differentiation of specific cell lineages, as well as conservation and maturation of stem cells in the stem cell niche. The vascular system and the intestinal mucosa are well-known tissues where Wnt and Notch pathway activities are found and cells are simultaneously exposed to opposing fates [43]. Frequently, cells react with a Wnt-ON/Notch-OFF state, probably due to downstream Wnt-dependent signaling inhibiting Notch as a standard [44]. There are several molecular mechanisms identified realizing a strong crosstalk between both important pathways expressed in the intestinal surface epithelium (Figure 3).

The multi-domain protein Disheveled, which is involved in activation of Wnt signaling, inhibits formation of the NICD activator complex by binding and reducing the CSL level within the pool of active transcription factors [37,38]. There are some data indicating that a molecular link exists between regulation of CSL and Her-2 activities [35,45]. The strong crosstalk of Wnt and Notch signaling via Disheveled and CSL could explain the conflicting findings of positive and negative Notch activities regarding Her-2. Inhibition of non-canonical Notch can also be achieved via Disheveled.

The important Wnt pathway component β-catenin also binds to Notch protein and is inhibited [38]. The physical interaction between both molecules suppresses the phenotypic effects of activated β-catenin in the Wnt signaling cascade. Frequently, the levels of Notch correlate inversely with those of β-catenin [38]. In *Xenopus leavis*, reduction of Notch is associated with translocation of β-catenin from intercellular junctions to the nucleus followed by ectopic activity of Wnt signaling. In cells of mammalian origin, accumulation of β-catenin is probably prevented by Notch. It has been shown that Notch interferes with the endolysosomal trafficking and degradation of β-catenin by Numb-mediated binding of activated β-catenin to Notch [46]. There is some evidence that the Notch-induced reduction of β-catenin-mediated Wnt activities is independent of the GSK3.

In addition to the Notch effect on Wnt, the Wnt pathway is able to modulate Notch signaling activities. For example, positive regulation of Notch is induced by direct binding and phosphorylation of the NICD with inhibition of its proteasomal degradation [38]. In CRCs, the Notch pathway effector Hath1 is negatively regulated by the Wnt pathway, resulting in a reduced MUC4 expression [47]. Recently, Notch-2 was identified as a novel target for β-catenin-dependent Wnt signaling and evidence is given that additional Notch-pathway-related genes might be transcriptionally regulated by the Wnt cascade [48]. In general, the Notch-Wnt crosstalk is probably essential to keep epithelial cells in a proliferative state, which is dysregulated and imbalanced in intestinal carcinogenesis. Loss of nuclear Hes1 expression is frequently found in CRCs of the right hemi-colon and is associated with *BRAF*V600E mutation, MSI status, and a poor prognosis [49].

Phosphorylation of the C-terminal domain in the NICD is mediated by the cyclin C cyclin-dependent kinase 8 complex and GSK3, which is assumed as another significant crosstalk between Wnt and Notch signaling [34,36]. In addition, *CCND1* was shown to be an important target gene in the Wnt cascade, as well as in the Notch signaling pathway. 

In summary, the main avenues of the Wnt-ON/Notch-OFF crosstalk is performed by the Wnt-associated protein Disheveled, which inhibits Notch and RBP, and the GSK3, which physically bind and phosphorylate the intracellular domain of two Notch paralogues. The inhibition of β-catenin by Notch is probably important in translating the Wnt-OFF/Notch-ON state [37]. These data suggest that Notch signaling is essential in the regulation of cell fate decisions in the intestinal epithelia, controlling the relative production of secretory versus non-secretory cells. At the crypt bottom and the proliferative intestinal niche, the crosstalk of Wnt and Notch signaling is crucial in the master switch from cell proliferation and suppressed differentiation into non-proliferative and differentiated intestinal cell lineages.

## 5. Targeting Wnt Signaling via Notch in Intestinal Mucosa

The inverse association of Notch and Wnt levels with the rapid switch between the Wnt-ON/Notch-OFF state and the Wnt-OFF/Notch-ON state gives a solid molecular basis to target Wnt signaling via Notch. The strong link and crosstalk between the Wnt and Notch signaling pathways is addressed with the term “Wntch” [37]. In the intestine, competition of both pathways is essential in differentiation and maturation of intestinal surface epithelia. Notch promotes the adoption of the absorptive enterocyte cell fate, while Wnt signaling is essential for differentiation of secretory cells, such as goblet cells, Paneth cells, and enteroendocrine cells [1,50,51]. The Wnt signaling with establishment of a Wnt gradient promotes the self-renewal of intestinal stem cells and configuration of the intestinal crypts [52,53]. In the case of Notch blockade, intestinal stem cell function is disturbed by de-repression of Wnt signaling activities with aberrant expression of pro-secretory genes (Figure 2). In order to modulate Notch activities, some inhibitors, such as MK-0752 and the azepine DAPT (GSI-IX), are used [54].

**Figure 4:**
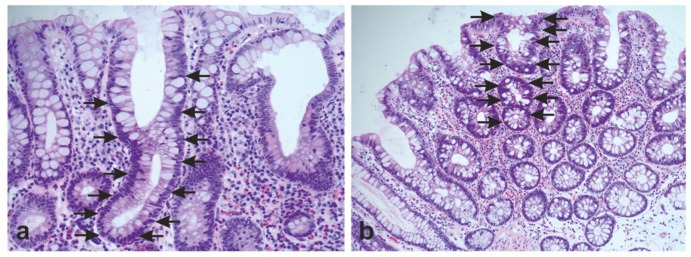
Morphological features of adenomatous polyps in the human large intestine. (**a**) “Bottom-up” morphology characterized by ascending cells with nuclear atypia (arrows indicate atypical cells) and normal plateau. (**b**) “Top-down” morphology with accumulation of atypical cells at the mucosal plateau (arrows indicate atypical cells), but normal cell composition, including goblet cells at the crypt basis. Original magnification: 200×.

Establishment of adenomas is assumed as an important step in intestinal carcinogenesis. There are two basal concepts to describe the intraepithelial propagation of neoplastic cells. In the “top-down” model, the cells become neoplastic by re-acquiring stem cell-like properties. They are not able to perform programmed cell death at the top (mucosal plateau) and propagate into the crypts followed by lateral migration. In the “bottom-up” model, the contrary is described. The stem cells at the crypts acquire genetic alterations followed by clonal expansion with migration upwards to the mucosal plateau (Figure 4a,b).

An important driver in the development of the neoplastic cell autonomy is Wnt signaling. The *APC* is a hotspot for mutational transformation of Wnt signaling in an active state, resulting in adenoma and adenocarcinoma. The heterogeneity of Wnt in colorectal adenocarcinomas is addressed with aggressive Wnt^high^ and moderate Wnt^low^ tumor cells [55]. It has to be stressed that the Wnt^high^ status of carcinomas is not exclusively due to the mutational type or load of the Wnt cascade in the tumor cells. Inflammatory macrophages in the surrounding stroma are important sources of the Wnt7B ligand. The secretory molecules Wnt7B and Wnt3 contribute to maintain the Wnt^high^ status [56,57]. Macrophages are one of the most abundant immune cells in the microenvironment of solid tumors and a strong correlation exists between the density of macrophages and poor outcome of different types of carcinomas [58]. There is experimental evidence that tumor-associated macrophages (TAMs) are mainly derived from Ly6C^high^ CCR2^+^ inflammatory monocytes that proliferate and transdifferentiate, but the contribution of tissue-resident embryo-derived macrophages to TAM populations is under discussion [59].

It is assumed that miRNAs control cancer development in a traditional manner by regulating important signaling pathways and growth factors. In addition, recent developments and discoveries indicate non-conventional mechanisms of cancer regulation by stem cell reprograming via a regulatory network consisting of miRNAs, as well as Wnt, Notch, and Hedgehog signaling pathways, all of which are involved in controlling functions of cancer stem cells [60]. Regulatory miRNAs are probably involved in the pathogenesis of CRCs through dysregulation of the APC and the Wnt/β-catenin signaling. In particular, miR-494 was identified as a prognostic marker and therapeutic target controlling CRC cell proliferation due to induction of the Wnt signaling pathway [61].

Caspase 8 (Casp8), an aspartate-specific cysteine protease (formerly known as FLICE), is synthesized by several cell types, including enterocytes. The protease is of high importance in regulation of different biological functions. It can promote cell survival activities under certain circumstances, but predominantly it has an important and central function in different forms of cell death, mainly assisted by FADD, RIPK1, and RIPK3, as well as cFLIP_L_. Casp8 is involved in the balance of anti-inflammatory apoptotic cell death (including generation of apoptotic bodies and their removal by phagocytic cells) and induction of pro-inflammatory necroptosis (i.e., rupture of plasma membrane and subsequent release of cellular content into the microenvironment) [62,63,64]. The complex function of Casp8 in intestinal mucosa includes regulation of TNF-α-induced epithelial necroptosis [65] with inhibition of RIPK3-dependent necrosis [66] and is involved in the functional complementation between FADD and RIPK1 [67]. Importantly, classic apoptosis is rare in the normal intestinal epithelium. Evidence is given that the replacement of epithelial cells at the tip of the CVA is functionally independent of an initiation of apoptotic cell death and has been referred to as homeostatic cell shedding [68,69].

In the intestine, disruption of normal cell death pathways is highly linked to the pathogenesis of different inflammatory and non-inflammatory diseases as well as carcinogenesis. In particular, the *Casp8* polymorphism 652 6N del is associated with a reduced cancer risk, especially concerning CRCs [70], but in vivo high expression of Casp8 in CRC is associated with poor survival [71]. This finding suggests a growth advantage of highly expressing Casp8-CRC tumor cells. Since Casp8 interacts with focal adhesion kinase and calpain2 and catalytic activities of Casp8 are not required for enhanced cell migration, some evidence is given for more complex Casp8 functions, which probably assist fundamental signaling pathways, such as the Wnt, Notch, and so-called Wntch network. 

**Figure 5:**
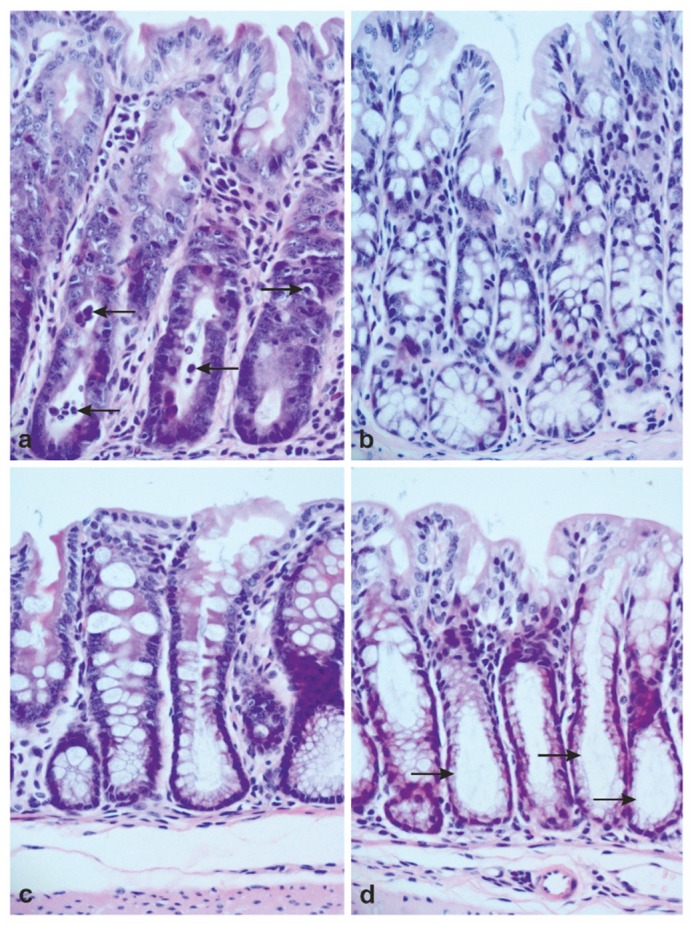
Targeting Wnt via Notch in a model of Casp8 deletion. (**a**) Severe cell death in the large intestinal mucosa of Casp8^f/f^ villinCre+ mice with deletion in Casp8 in intestinal epithelial cells (Casp8^Δint^). Arrows indicate examples of apoptotic bodies. (**b**) Typical features of normal large intestinal mucosa in Casp8^f/f^ villinCre- control littermates (Casp8^f/f^) with a high number of goblet cells. (**c**) Restoration of goblet cells in the large intestinal mucosa of Casp8^Δint^ animals treated with the Notch inhibitor dibenzazepine DBZ. (**d**) Large intestinal mucosa of Casp8^f/f^ mice after DBZ treatment with mucostatic features at crypts (arrows). Original magnification: 200×.

In intestinal mucosa, targeting of Wnt via Notch was addressed in mice with deletion of Casp8 in intestinal epithelial cells (Casp8^Δint^). Previous data indicate that these mice may serve as a model of Notch activation [72]. In Casp8^Δint^ animals, intestinal inflammation and severe injury of the epithelial barrier with reduction of Paneth cells and goblet cells in the small intestine and strong cell death with reduction of goblet cells in the large intestine (Figure 5a,b) were found. The diminished number of secretory cells was associated with a strong increase of Notch activity with elevated Hes1 mRNA levels. The expression level of Math1, the repressor of Hes1, was diminished. In line with these observations, the genetic deletion of *Casp8* was associated with rapid generation of cells failing to differentiate in secretory cell lines and hampered migration along the CVA and CPA [73]. However, the typical phenotype of crypt-progenitor cells with re-localization of β-catenin into the nucleus was not found, but the expression of the functionally important Wnt target gene *Leucine-rich repeat-containing*, *G-protein-coupled receptor 5* (*LGR5*) was seen. *LGR5* belongs to about 80 genes that are regulated by Wnt signaling. Its expression is restricted to crypt bottoms and particularly found in crypt base columnar cells that are interspersed between the Paneth cells. The Lgr5 positive cell type represents the long-lived stem cells of the small and large intestinal mucosa [74,75].

Dibenzazepine (DBZ) is a chemical compound characterized by two benzene rings fused to an azepine group. DBZ is well established as an inhibitor of γ-secretase (GSI), which is essential for activation of canonical Notch signaling (Figure 2). In Casp8^Δint^ mice, DBZ treatment was highly efficient in inhibiting Notch activities. The pharmacological inhibition of Notch was associated with a decrease of Hes1 mRNA expression and increased Math1 levels. These regulative effects were found throughout the small and large intestine, but were most visible in the ileum. Morphologically, the number of goblet and Paneth cells was dramatically increased in the small intestine and an increased number of goblet cells was found throughout the colon, indicating an activated Wnt signaling after pharmacological blockade of Notch in a Casp8^Δint^ background (Figure 5c,d). Interestingly, the Lgr5 mRNA expression levels were heterogeneous in the model. The expression of Lgr5 was increased in the small intestinal mucosa of Casp8^Δint^ animals with Notch activation. After DBZ treatment a diminished expression of Lgr5 was found that did not correlate with the establishment of a Wnt-ON/Notch-OFF signaling dependent secretory mucosal phenotype. Based on these data it could be speculated that a Casp8 target with relevance for the Wnt-ON/Notch-OFF state regulating the Wntch may exist, or DBZ, per se, disrupts function and differentiation of progenitor and stem cells.

## 6. Wntch Targeting and Clinical Trials

In the Wntch, the interacting molecules and their activities are promising targets for small molecular drugs and immune checkpoint inhibitors. One important target in the Notch pathway is the enzyme γ-secretase (Figure 2). Pharmacological inhibition of this molecule is associated different models with an encouraging inhibition of the Wnt signaling and tumor regression. The small molecule MK0752 is a γ-secretase inhibitor and is used in phase I and II clinical trials. In the breast cancer study NCT00645333, MK0752 is used in combination with docetaxel [76], whereas the combination with gemcitabine is tested in the pancreatic cancer study (NCT01098344) [77]. Another small molecule, RO4929097, is used in phase II clinical trials for the treatment of colorectal (NCT01116687) [78] or pancreatic cancer (RO4929097) [79]. For the treatment of lymphoid malignancies or solid tumors, other inhibitors, such as BMS-906024, a pan-Notch inhibitor, or anti-Notch specific antibodies, are being tested in phase I trials [16]. Recently, the benefit of PF-06650808, an anti-Notch 3 antibody-drug conjugate, was shown in a dose-escalation phase I study in patients suffering from breast cancer [80]. It has to be stressed that the tremendous crosstalk between the Wnt and Notch pathways, including their respective long non-coding RNAs, offers promising opportunities in future drug discovery [81]. However, the complexity of the Wntch sets the limits.

## 7. lncRNAs as a Promising Tool Targeting the Wntch in Intestinal Cancer

Long non-coding RNAs (lncRNA) are defined as non-coding RNAs that are more than 200 nucleotides in length. This type of RNA is heterogeneous and shows no sequence conservation. It is assumed that the expression of some lncRNA types is restricted to certain developmental stages with a distinct subcellular localization [82]. Notch and Wnt pathways have been identified in ISC and CRC stem cells. There is accumulating evidence that in both cellular systems lncRNAs may be important in regulation stemness and acquisition of an aggressive behavior [83]. For example, FOXD2-AS1, coded on chromosome 1p33, with a transcript length of 2527 nucleotides, exhibits aberrant expression in various types of cancer and was identified as a promoter of CRC progression via Notch [84]. The expression of another lncRNA, FAM83H-AS1, was investigated in CRCs. Similar to FOXD2-AS1, FAM83H-AS1 up-regulates Notch and is clearly involved in carcinogenesis, particularly in development of CRC [85]. In addition, other lncRNAs originating from the amplified cancer-associated chromosome 8q24 region (a so-called gene desert) probably regulate the Wnt signaling cascade [86,87]. The class of CCAT lncRNAs, especially CCAT1, is thought to be a basic signaling network in CRC development. The strong association of increased CCAT1 expression levels in CRCs predicts this lncRNA type to be an important prognostic biomarker in CRC [88]. Interestingly, expression of CCAT1 correlates with the CRC response to JQ1, a chemical inhibitor of the BRD4 oncogenic cascade in CRC [89]. In conclusion, targeting the Wntch via lncRNA species, e.g., FOXD2-AS1, may be a promising and effective strategy for CRC treatment and disease control. 

## 8. Conclusions

Canonical, as well as non-canonical, Wnt and Notch signaling are fundamental molecular pathways. A plethora of experimental evidence exists that Wnt and Notch activities are regulative elements for inverse cell fate decisions including cellular proliferation, differentiation, trans-differentiation, and stem cell behavior. An intensive crosstalk between the two pathways at different levels is found, which is important to regulate cell fate decisions. For example, regulatory proteins of the Wnt pathway are simultaneously involved in regulation of Notch signaling and expression of some genes is addressed by both signaling cascades. The strong interaction of Wnt and Notch, the Wntch, appears as an interesting tool for pathway targeting in non-neoplastic intestinal diseases and colorectal cancer.
